# 6-Fold path to self-forgiveness: an interdisciplinary model for the treatment of moral injury with intervention strategies for clinicians

**DOI:** 10.3389/fpsyg.2024.1437070

**Published:** 2024-11-25

**Authors:** Michele J. DeMarco

**Affiliations:** California Institute of Integral Studies, San Francisco, CA, United States

**Keywords:** moral injury, forgiveness, self-forgiveness, counseling, interventions, relationships, PTSD-posttraumatic stress disorder

## Abstract

Conscience is the indestructible core of one’s personal identity and their sense of agency in the world. When it passes judgment against them, it generates inner conflict (i.e., moral injury). At its core, moral injury is about trust and sacred relationships, particularly the loss of safe connection with self, society, God/Divine/a Higher Power, and the world. The clash between a person’s conscience and overwhelming existential or psychospiritual experiences, which uniquely defines moral injury, alienates them from life-sustaining relationships. Healing requires more than reordering fractured belief systems. Reestablishing bonds of self-worth, trust, and life-sustaining relationships are essential. This paper presents the 6-Fold Path to Self-Forgiveness (6-FPSF), an interdisciplinary, narrative-based healing writing process for the treatment of moral injury, particularly self-induced moral injury. Self-forgiveness has been associated with psychospiritual and relational well-being. The protocol draws upon theoretical literature, evidence-based psychological interventions, spiritual-oriented practices, creative arts, and somatic exercises for mental health counseling and spiritual/religious ministration. In addition to describing the 6-component therapeutic model, the author offers intervention strategies for clinicians.

## Introduction

Researchers are still debating whether *moral injury* (MI) is a distinct aspect of trauma exposure, separate from *posttraumatic stress disorder* (PTSD). No consensus definition of MI has yet emerged; however, there is increasing agreement that MI happens when a person’s core moral foundations are violated in high stakes situations such as participating in, witnessing, or failing to prevent acts that transgress their core values and sacred beliefs. This violation recasts the way people see themselves, others, and the world and causes changes in behavior that signal a loss of trust, connection, self-worth, and meaning (Bremault-Phillips et al., 2022; Currier et al., 2015).

MI has been associated with feelings of shame, guilt, grief, despair, betrayal, alienation, condemnation, helplessness, and powerlessness (Barnes et al., 2019; Litz et al., 2009). Rumination, excessive blame, and distrust are common, as are emotional dysregulation, lack of self-acceptance, and negative self-appraisals (Bremault-Phillips et al., 2022). Increased risk of depression, emotional shut-down/numbing, substance abuse, self-handicapping activities, and risky behavior are also common (Barnes et al., 2019; Farnsworth et al., 2014).

The clash between a person’s conscience and overwhelming existential experiences, which uniquely defines MI (Graham, 2017; Hodgson and Carey, 2017) can result in an acute loss of one’s faith and erosion of meaning and purpose (Farnsworth et al., 2014; Usset et al., 2020), questioning of goodness and the world order (Bremault-Phillips et al., 2022), and a shattering of the Sacred. The Sacred is understood as that which is held with reverence, considered holy or sacrosanct, including God/Divine/a Higher Power, the transcendent, and ground of being (Wulff, 1997). For many people with MI, there is a profound feeling of being divided in their soul, which alienates them from life-sustaining relationships (Brock and Lettini, 2012; Graham, 2017).

Thus, MI can be viewed as multidimensional in nature, requiring an interdisciplinary, integrated approach to healing. Kinghorn (2012) has suggested that effectively, all forms of injury or offense—psychological, emotional, biological, social, behavioral, relational, and spiritual—have an element of moral trauma such that a person may question their own moral worth. Similarly, others (Bremault-Phillips et al., 2022; Currier et al., 2015; Hodgson and Carey, 2017; Litz et al., 2009; Pernicano et al., 2022; Usset et al., 2021) have implied that MI’s unique psychospiritual phenomenology would be best served with a holistic approach.

### Forgiveness as moral repair

Forgiveness is a complex neurocognitive, affective, and spiritual process that is garnering increasing attention across disciplines (Bremault-Phillips et al., 2022; Haikola, 2023). Like MI, no consensus definition of forgiveness has been found, yet various conceptions and essential components have emerged. The American Psychological Association (2024) identifies intentionally transforming deeply held negative feelings such as anger, resentment, vengeance, and injustice that keep people stuck in unforgiveness, and moving them toward a form of grace—understood as undeserved goodwill, compassion, generosity, and benevolence—as being fundamental to forgiveness. There is consensus that constructs such as excusing, pardoning, condoning, and forgetting should be distinguished from forgiveness (Rye et al., 2001; Strelan and Covic, 2006), whereas the construct of reconciliation remains unclear: some researchers agree that reconciliation should also remain distinct (Strelan and Covic, 2006), while others have suggested reconciliation is the desired endpoint for forgiveness (Fitzgibbons, 1986; Hargrave, 1994; Pollard et al., 1998). The question of where forgiveness ends remains unanswered. In addition to reconciliation, some researchers have argued that the endpoint of forgiveness is the absence of negative cognitions, affect, and behavior (Gordon and Baucom, 1998; Thompson et al., 2005). Others suggest it is when a voluntary gift is made to release the offender from obligation (Exline and Baumeister, 2000). McCullough et al. (2000) posited that forgiveness ends with the transformation of a motivational state, whereby avoidance or retaliation gives way to positive motivation. Forgiveness had once been considered primarily intrapersonal in nature (Baumeister et al., 1998). More recently, researchers have asserted that forgiveness is, at its core, relational (Buhagar, 2021; Griffin et al., 2015) and occurs on a continuum from hostility, estrangement, and unforgiveness to complete forgiveness, friendliness, and relationship restoration (Forster et al., 2020; Bremault-Phillips et al., 2022).

Much research has shown that the benefits of forgiveness are far-ranging, such as positive psychological, mental, spiritual, and relational health (Long et al., 2020; Toussaint et al., 2001; Worthington et al., 2007); decreased anxiety, depression, and stress; increased self-esteem, personal growth, hope, and wholeness; and reciprocal forgiveness between others and oneself (Cornish and Wade, 2015; Kim et al., 2022; Worthington and Langberg, 2012). Thompson and Korsgaard (2019) have suggested that forgiveness facilitates relationship resilience where the relationship becomes stronger than it was prior to the offense.

Significant research has also shown that forgiveness is particularly effective when attending to issues common in MI. These include moral emotions such as guilt, shame, and anger; self-worth, regret, and blame, the loss of meaning, purpose, value, connection, resilience, and transcendence (Haidt, 2003; Harris et al., 2006; Pargament et al., 2013; Wade and Worthington, 2003); and rumination (de la Fuente-Anuncibay et al., 2021). Forgiveness is positively related to the development of empathy and perspective (Konstam et al., 2011; McCullough et al., 1997).

Forgiveness is not only something that happens in one’s mind but also in one’s body (Haikola, 2023). Forgiveness has been related to smiling, improved blood pressure, slower heartbeat and lower risk of heart attack, reduction of pain, cholesterol levels, and sleep, calmer physiology such as increases in parasympathetic activity, and more relaxed facial muscles (da Silva et al., 2017; Kim et al., 2022; Lawler-Row et al., 2011). Forgiveness also has nuances on sensory and embodied levels (Haikola, 2023). Embodiment concerns the way in which a person’s emotions, sensations, thoughts, and behaviors interact with the world (Meier et al., 2012). This connection between bodily experiences and psychological processes informs one’s overall lived experience, which affects how a person orients in life and narrates their life story (McAdams and Dunlop, 2022).

Humans understand their lives by constructing narratives (i.e., stories) out of their experience (Baumeister and Newman, 1994). Stories provide a way for people to make sense of themselves, others, and the world and to prioritize them in their mind. Through story a person forms and examines what they believe is true, and then they set those truths against other truths. Researchers (Freedman and Combs, 1996; White and Epstein, 1990) have suggested that much of a person’s suffering is not caused by the factual events of what happened to them; rather, by the stories they tell themselves about what happened. Baumeister and Newman (1994) posited that there are four needs for making sense of experiences as guiding narrative thought:

“First, people interpret experiences relative to purposes, which may be either objective goals or subjective fulfillment states. Second, people seek value and justification by constructing stories that depict their actions and intentions as right and good. Third, people seek a sense of efficacy by making stories that contain information about how to exert control. Fourth, people seek a sense of self-worth by making stories that portray themselves as attractive and competent. Within this framework, narratives are effective means of making sense of experiences.” (p. 676)

Thus, stories help to provide internal coherence, which enables a sense of harmony with a person and their place in the world. Moral transgressions fracture this sense of internal coherence and, alternatively, cause internal dissonance (Bauer et al., 1992; Pernicano et al., 2022). The *principle of symptom of coherence* (Chamberlin, 2023) suggests that whatever manifestations of pain or suffering a person experiences are not a disease or a pathology. Rather, they are adaptive, protective, mechanisms, based on mental models that one has developed throughout their lives, such as biology, genes, attachments as children, habits, mores and values, associations, belief systems, and the nervous system.

Emotional memories of negative events, such as moral transgressions, are particularly impactful. This is because humans are programmed for protection to experience negative emotions more rapidly and more intensely and to elicit more prominent responses than positive ones (Baumeister et al., 2001; Brosschot and Thayer, 2003); this is referred to as *negativity bias* (Vaish et al., 2008). For instance, negativity bias affects recall, causing a person to focus on transgressions more than compliments; dwell on painful or traumatic experiences more than pleasant ones; and direct attention faster to negative information than positive (Chen and Lurie, 2013; Wisco et al., 2014). While emotions become more nuanced and differentiated with time and experience, forgiveness still can be difficult, because, as previously mentioned, moral transgressions elicit intense emotions, which can shape emotional memories, personal narratives, and grievances stories (Haikola, 2023).

### Self-forgiveness as a way of being

Approaches to forgiveness may be particularly helpful for healing from MI. Enright’s (1996) forgiveness triad includes (a) forgiving others, (b) receiving forgiveness from others, and (c) self-forgiveness, which fits nicely with types of MI, those being self-induced, other induced, and self/other induced (Barnes et al., 2019; Nieuwsma et al., 2022). While past research has centered on studying other-related forgiveness, less has focused on self-forgiveness (Enright, 1996; Long et al., 2020). With the shift from a purely intrapersonal conception of forgiveness (including self-forgiveness) to incorporate an interpersonal conception, understandings of the components of self-forgiveness have likewise shifted from those that are self-focused emotional, motivational, and behavioral to ones that also include reparative behaviors to the person or entity that was wronged and a recommitment to values (Bremault-Phillips et al., 2022; Cornish and Wade, 2015).

Like other-related forgiveness, including with the Sacred, self-forgiveness is associated with psychological, mental, and spiritual health; for instance, meaningful personal growth (Cornish and Wade, 2015; Hsu, 2021); perceived quality of life (Romero et al., 2006), satisfaction with life (Thompson et al., 2005), self-trust (Walker and Gorsuch, 2002), self-esteem, positive emotions and a lack of shame (Worthington et al., 2005); benevolence towards the self (McConnell, 2015), increased meaning, purpose, and coherence (Bauer et al., 1992), increased value reaffirmation (Wenzel et al., 2021) and self-acceptance (McGaffin et al., 2013). People who practice self-forgiveness also have more positive attitudes (Cornish and Wade, 2015), healthier relationships (Pierro et al., 2018), and higher levels of success, focus, and concentration (BeWell Stanford, 2019). Self-forgiveness also serves as a defense against conditions such as anxiety, depression, PTSD, and neuroticism (Pierro et al., 2018), mood disorder (Friedman et al., 2007), self-punishment and self-condemnation (BeWell Stanford, 2019; Friedman et al., 2007); and rumination (Thompson et al., 2005; Lucas et al., 2010).

Unlike other-related forgiveness, researchers (Enright, 1996) have suggested that reconciliation is always linked to self-forgiveness. “One does not offer only an affective or cognitive response to oneself, but truly cares for oneself as a member of the human community … one does not remain alienated from the self” (p. 9).

Definitions of self-forgiveness include “a willingness to abandon self-resentment in the face of one’s own acknowledged objective wrong, while fostering compassion, generosity, and love toward oneself” (Enright, 1996, p. 9); “a set of motivational changes whereby one becomes decreasingly motivated to avoid stimuli associated with the offense, decreasingly motivated to retaliate against the self (e.g., punish the self, engage in self-destructive behavior), and increasingly motivated to act benevolently toward the self” (Hall and Fincham, 2005, p. 622); a positive attitudinal shift in the feelings, actions, and beliefs about the self, following a self-perceived transgression or wrongdoing committed by the self (Pierro et al., 2018); “the process of neutralizing a stressor that has resulted from a perception of an interpersonal hurt” (Strelan and Covic, 2006, p. 1076); and “how one views oneself and aims to free the self from guilt or shame by accepting responsibility for having violated socio-cultural and S/R (i.e., Spiritual and Religious) values and beliefs” (Bremault-Phillips et al., 2022, p. 3). Cornish and Wade (2015) define self-forgiveness as “a process in which a person a) accepts *responsibility* for having harmed another; b) expresses *remorse* while reducing shame; c) engages in *restoration* through reparative behaviors and a recommitment to values; and thus d) achieves a *renewal* of self-respect, self-compassion, and self-acceptance” (p. 97). Some researchers (Griffin et al., 2015; Davis et al., 2015) have gone further to conceptualize self-forgiveness as a coping strategy embedded within an adapted stress-and-coping model but differentiate it from other coping models that focus on either an immunity approach (i.e., denying culpability and excusing oneself from blame) or a punitive approach (i.e., self-punishing, self-worth-denying atonement). In this model, self-forgiveness can be understood through the lens of transformation in that a transgressor significantly modifies the view of themselves to a new self-concept that integrates personal responsibility for an offense with a prior sense of self-worth.

As of this writing, no program for forgiveness, particularly self-forgiveness, existed that integrated all the afore-mentioned content elements necessary for addressing the multidimensional, embodied nature of MI: (a) self- and other-focused; (b) psychological, emotional, biological, cognitive, behavioral, relational, and spiritual intervention approaches; (c) transformation of self-concept that leverages a *coherent sense of time* (DeMarco, 2024a)—that is seeing oneself fully in the past, present, and future—which does not bring a person merely “back” to a prior sense of self-worth, rather re-creates a new and integrated self-concept and life narrative; (d) includes the concept of reconciliation; (e) is desire-based (i.e., focuses on renewed meaning, purpose, value, connection, resilience, and transcendence, rather than on symptom reduction and reordering fractured belief systems); and (f) incorporates somatic psychology and embodied therapies, as they have been shown to be particularly successful in treating trauma (Brom et al., 2017; Levine, 2010; van der Kolk, 2014). Further, I suggest that self-forgiveness should be approached as a “way of being,” that is a mode of existence, which is more than an attitude, mood, or set of behaviors; it is a holistic felt sense and ongoing process, in this case a process of moral resilience (Rushton, 2016), that does not culminate as a final point on a spectrum; instead, abides sustainably in conscience, character, choice, commitment, community, and contribution.

### Introduction to the 6-fold path to self-forgiveness (6-FPSF)

The 6-FPSF is an interdisciplinary, narrative-based healing writing process for the treatment of MI, particularly self-induced MI, that incorporates the contents above. The protocol draws upon theoretical literature, evidence-based psychological interventions, spiritual-oriented practices, creative arts, and somatic exercises for mental health counseling and spiritual/religious ministration. Among these include *embodied disclosure therapy* (EDT; DeMarco, 2022). EDT is a brief therapeutic approach that integrates body awareness into the writing process, something that appears to have not been used in other writing therapies or as a primary mode of treatment for MI. EDT was purposefully developed to overcome the challenges of *expressive writing* and other exposure-based writing therapies, such as *written exposure therapy* (WET) that use fast-paced, explosive, “let-it-all-go” writing, causing agitation, rumination, overwhelm, or shut down in certain populations. Like WET, EDT exposes a person to emotionally and psychospiritually challenging material and blocks avoidant responses but further integrates narrative emplotment into the instructional set to address the issue of meaning and coherence (or lack thereof) that is unique to and necessary for healing from MI. The 6-FPSF also leverages insights from narrative therapy (Freedman and Combs, 1996; White and Epstein, 1990) such as re-authoring identity, internal stories and external conversations, and the construction and deconstruction of meaning. It is well documented that narrative disclosure, that is recalling and renegotiating a traumatic story, is considered an important component of the healing process (Kearney, 2007; Marriott et al., 2016). Insights are further drawn upon from spiritually-focused approaches (Hodge, 2005; Miller, 1999; Richards and Barkham, 2022; Walsh, 2009), Adaptive Disclosure Therapy (ADT; Litz et al., 2016), Somatic Experiencing (Levine, 2010), and psychedelic integration models (Frymann et al., 2022; Siegel, 2010).

The goals of treatment include (a) *reckoning* the transgression and harm; (b) expressing *remorse* after metabolizing difficult truths and resulting emotions, feelings, and sensations; (c) *reconciling* the injurious experience by putting an end to pain and hostility towards oneself and by bringing the self into harmony with others and the world; (d) *rectifying* what has been damaged, and restoring right relationships through reparative actions and adopting new ways of thinking, engaging, and connecting; (e) *re-creation*, that is integrating the painful morally injurious experiences into a new concept of self and coherent life narrative; and (f) *remaining* true to the new morally resilient narrative as a way of being.

With MI, people seek self-forgiveness for actions they took (or failed to take) that violated their moral foundation, core values, or sacred beliefs, such as harming or killing, whether intentional, unintentional, or unavoidable; not being able to prevent a tragedy or negative outcome, inability to change a situation, accidents, mistakes, flawed behavior, betrayal, cruelty, carelessness, disloyalty, lying, cheating, falsifying information, neglect, abandonment, deprivation, discrimination, and oppression, among others. The purpose of the 6-FPSF is transforming self-condemnation, self-rejection, and self-estrangement that result from these experiences into self-worth, self-integration, and moral resilience.

Table 1 presents a two-phased, six component curriculum. During Phase 1, Coming Into Awareness, a person (i.e., the transgressor) (a) undertakes an intersubjective MI review—or an *examination of conscience*—whereby they confront and take account of the moral harm, assess their part in the transgression with “benevolent honesty” (DeMarco, 2024a), that is a kindness and gentleness with oneself, admit wrongdoing, and accept responsibility; (b) engages in “honest grieving” (DeMarco, 2024a), that is engaging painful memories, facing difficult truths, and metabolizing the resulting intense emotions, feelings, and sensations that can contribute to shame, guilt, and grievance narratives (Luskin, 2003); titration techniques are employed to help keep the person in their “window of tolerance” (Corrigan et al., 2011) and maintain homeostasis and rational thought necessary for meaning-making; and c) brings an end to hostility towards oneself and comes into harmony with others, God/Divine, and the world through “dynamic balance” (DeMarco, 2024a), that is maintaining a flexible, open, and accepting posture, and restoring a bond of trust, self-worth, and meaning. Phase 2, Soul Remaking, shifts the focus from internal self-awareness gained in Phase 1 to external self-expression by (a) invoking “positive intention” to actively fix what has been broken or injured and to restore right relationships; (b) affirming the sacredness—understood as that which is worthy, honorable, and deserving of respect—of self, and advancing a new story that integrates all aspects of the morally injurious experience with an expanded and coherent perspective; and (c) embracing self-forgiveness as a “way of being” that abides in self-worth, trust, and meaning.

**Table 1:** Mapping the 6-fold self-forgiveness model (6-FPSF).

Component	Description	Approach	Content
Phase 1: coming into awareness *(intersubjective review)*			
Reckoning	*Examination of conscience*	Benevolent honesty *(Cognitive)*	Intentional move toward self-forgiveness; understand what moral identity uniquely looks like. Transgressor confronts and takes account of harm, assesses their part in the transgression, admits wrongdoing, and accepts responsibility.
Remorse	*Metabolize difficult truths and resulting emotions*	Honest Grieving *(Emotional)*	Appreciate the impact of the injurious experience—the harm caused, values violated, perspective shifts, relationship alterations or severance. Identify “core wounds.”
Reconcile	*End of hostility with oneself. Come into harmony with others and the world*	Dynamic balance *(Psycho-spiritual)*	Address internal dissonance. Overcome self-deception; understand and accept limits of responsibility; avoid perceived and excessive responsibility. Honor the past. Integrate painful memories into life’s larger story. Make oneself available to a “moment of grace.”
Phase 2: soul remaking *(external expression)*			
Rectify	*Fix what’s been broken; restore right relationships*	Positive intention *(Behavioral)*	Voice nature of transgression (as it is safe to do so). Receive response with benevolent honesty. Make a gift/ritual if making amends is not possible. Find new or renew purpose. Recommit to values. Explore harmful attitudes and beliefs. Determine steps to avoid future harm. Implement new boundaries.
Re-creation	*Integration of painful experiences into a new and coherent narrative*	“Kintsugi” *(Embodied)*	Move from self-estrangement to being “at home” the world. Integrate painful experiences as sources of wisdom and guidance. Embrace self-acceptance, self-compassion, self-mastery, and self-expression. Advocate for integrity and authenticity. Attend to personal growth. Honor one’s intrinsic self-worth as a *Sacred Self*.
Remain	*Self-forgiveness as a way of being*	Moral resilience *(Embodied)*	Abide in conscience, character, choice, commitment, community, and contribution. Appreciate the journey to here and beyond. Accommodate twinges of negative emotion and reset with finding meaning, purpose, and connection. Attune values ongoing. Practice “ethical embodiment.” Cultivate a moral vocabulary, imagination, attitude, and coherent character, as well as a dynamic moral posture. Live in self-worth, trust, and meaning.

The framework for the 6-FPSF consists of two writing and integration sessions per week, spaced 2 to 3 days apart, for 6 weeks, lasting 60–90 min, each facilitated by a practitioner who is familiar with or has specialized training in traumatic stress and preferably psychospiritual concerns and practices. The practitioner should also have an empathetic presence, self-awareness, understanding, and integrity who can support, rather than direct the process (Buhagar, 2021; Frymann et al., 2022). For clients who have engaged in some form of trauma-focused care or processing traumatic emotional content, they may move through the process more quickly than those who have not. Alternatively, because self-forgiveness is complex and requires an arduous process of psychological, emotional, mental, relational, and spiritual engagement (Nash and Litz, 2013), some clients may benefit from increasing the number of sessions (e.g., from 12 to 24) to provide more space for writing, reflection, and integration; integration in this context is understood as a means of making sense and finding significance out of the writing experience, discovering insights gleaned during the session, and applying them to the process of self-forgiveness going forward (Frymann et al., 2022; Gandy et al., 2020; Guss et al., 2020). The pace and timing of genuine self-forgiveness is not universal, and people need to be ready and willing to forgive (Bremault-Phillips et al., 2022). Practitioners and clients are encouraged to focus on engaging each step of the process with intention, patience, and commitment, rather than moving swiftly to a desired endpoint.

As with other writing therapies, clients are advised not to worry about grammar, spelling, or page count during writing and should write without attachment to the outcome or audience (i.e., writing is only for its author). At the beginning of each session, clients attune their body and mind to their surroundings and to a sense of safety and presence, as possible. They also set an intention for the session related to one of the model’s six components (i.e., reckoning, remorse, reconcile, rectify, re-creation, and remain). Intention setting provides a direction and purpose for the therapeutic process and maximizes its effectiveness (Frymann et al., 2022). Intentions serve as a guide for navigating feelings, thoughts, emotions, and sensations; clarifying personal values and values-congruent actions; and facilitating exploration, self-reflection, self-awareness, and self-discovery (Guss et al., 2020). Clients then ready themselves for writing with a grounding exercise and begin the writing process.

During each of the sessions, instructions and writing prompts are provided by the practitioner. They also answer any clarifying questions that the client has. Writing can be done with or without the presence of the practitioner. In WET (Sloan et al., 2018) and EDT (DeMarco, 2022), the practitioner exits the room during writing (unless it is clinically unadvisable to leave the client alone), leaving a printed copy of the writing instructions and prompts for that session with the client so that they can refer to them. In some instances, clients prefer the practitioner to remain in the room, which is acceptable, so long as the practitioner is not a distraction from writing.

Because trust (or the fracturing of trust) is central to MI, the locus of trust, during writing, is more internally situated, that is with the client using the writing process to rebuild trust with themselves, others, and the world. In this way the writing process itself acts as the “therapist” or practitioner. Therapeutic attunement, that is the nonlinear process in which therapists track the moment-to-moment changes in the somatic, emotional, and energetic rhythms of the client, and the intersubjective relationship that exists between them (Feiner-Homer, 2016), is embedded in the writing process and is inner directed and centered on the client-writing relationship. In this way, the role of the practitioner, during writing, is more of a trustworthy steward or a reliable shepherd for the integrity of the writing process.

After 30 min, the practitioner ends the writing session and inquires whether the client experienced any difficulties during the session and addresses any problem or concerns that may have arisen. The practitioner also asks clients to rate their distress and social connection levels, respectively, on a scale from 0 to 10 and then begins the integration process.

The integration process includes reflection and application (Frymann et al., 2022; Phelps, 2017). Through reflection, connections are made between the insights that grew out of the experience and the client’s life, attitude, and story; with application, insights can be put into action into daily life (Büssing et al., 2005; Guss et al., 2020). Integration may take place through individual and interpersonal means. Practitioner-guided integration is actively supported (Frymann et al., 2022; Phelps, 2017) by encouraging clients to make connections between intentions set pre-session, emergent meaning discovered during the session, and the respective components of 6-FPSF. If the process is in a group context, reflection can be supported through structured group sharing between participants (Frymann et al., 2022; Pernicano et al., 2022). Personal reflection can occur through additional writing, silent contemplation or meditation, being in nature, or other means of attunement to internal experiences and wisdom (Phelps, 2017) and can be done both in the session and/or between sessions. The session ends with the practitioner reiterating certain aspects of the psychoeducation.

Guidelines for the 6-FPSF include (a) select the trauma memory that is most salient, elicits the most symptoms, or is most representative of trauma exposure; (b) writing about the same event or experience during each session; (c) beginning each session by starting in a place of safety, connection, and calm; (d) writing at a slow to moderate pace; (e) the importance of delving into a person’s innermost emotions, feelings, and sensations as they relate to the traumatic experience; (f) fully engaging in the writing without breaks or distractions (e.g., listening to music, answering a call or text); and (g) the first session is typically 90 min, because it includes 30 min of psychoeducation about MI, forgiveness/self-forgiveness, and embodied reactions to trauma. Subsequent sessions are typically 60 min with a check-in at the beginning and end of each writing session, pre-session intention setting and readying/resourcing, and post-session integration to reflect on changes in the trauma memory, self-concept, or injury narrative.

### Program components and curriculum description

#### Phase 1: coming into awareness

*Coming Into Awareness* focuses on building self-awareness through interpsychic and somatic exploration.

##### Reckoning

Consensus opinion is that effective processing of a transgression must include the transgressor accepting responsibility for the violation (Cornish and Wade, 2015; Enright, 1996). Yet the literature (Cornish and Wade, 2015; Woodyatt and Wenzel, 2013a, 2013b) also proposes that this acceptance may threaten a person’s self-regard, status, and social inclusion and lead to threat avoidance or pseudo self-forgiveness. Pseudo self-forgiveness results when transgressors distort the impact of the harm, deny the wrongdoing, derogate blame, distract themselves from the offense with excuses, justifications, and rationalizations, or dissociate from the transgression altogether (Hall and Fincham, 2005; Woodyatt and Wenzel, 2013a,b).

Cornish and Wade (2015) have suggested that acceptance of responsibility involves “recognition of wrongdoing, acknowledgement that one could and should have done things differently, and a realization of one’s imperfection” (p. 97). This inventory supports the self-forgiveness literature (Bremault-Phillips et al., 2022; Enright, 1996; Griffin et al., 2017; Holmgren, 2012); however, I argue that for MI a fuller inventory of accepting responsibility must include a detailed narrative account of the transgressive events and resulting harms, as well as a clear understanding of one’s moral compass, core values, and sacred beliefs prior to the transgression. Otherwise, self-forgiveness risks more than pseudo self-forgiveness; it could result in a form of “cheap grace” (Bonhoeffer, 1995) whereby acceptance is in profession only and short-lived (Burkman et al., 2022). I also suggest that in some instances of MI it is the case that while one “could” have done things differently, it may not always be obvious that they “should” have; often a person must choose between conflicting wrongs. Thus, forcing that person into such a confession could do more harm than good; for instance, exacerbating symptoms, particularly threat avoidance, painful emotions, and resistance to the self-forgiveness process. I, therefore, present the first component of the 6-FPSF as *Reckoning*, a cognitively situated engagement that begins with an *examination of conscience*, including active intention to confront the violation, an account of the constituent elements of the transgressive events, acknowledgment of the harm caused—including relationship ruptures and affected relationship dynamics, an assessment of the person’s part of the transgression, an appraisal of one’s time-honored core values and sacred beliefs, admittance of wrongdoing, and acceptance of responsibility.

This form of personal testimony of the transgression and need for self-forgiveness is a vital first-step in the process. Such testimony must be honored as the person’s evident reality and approached with “benevolent honesty,” that is a transparent, unadulterated, unexaggerated, and undiminished internal exploration, yet undertaken with kindness, compassion, and gentleness towards oneself as aspects of the painful experience are uncovered and disclosed. Approaching *Reckoning* with benevolent honesty will help to lessen common reactions such as self-condemnation, threat avoidance, unnecessarily harsh self-appraisals, destructive impulses and cognitions, and distorted meanings (Buhagar, 2021).

Content activities for *Reckoning* include intention setting and pre-session grounding exercises, which focus on fostering self-awareness, presence, and courage; observing thoughts without judgment; and recalling events clearly and directly. Writing prompts include helping a person identify what, specifically, integrity looked like to them prior to their injurious experiences (i.e., “Awakening Core Integrity”), identify “Messages in Pain, and “Retelling the Past”—the latter of which is a “first draft” or narrative of one’s injury story (DeMarco, 2024b). Integration can include reflections on how the client is interpreting the morally injurious experience vis-à-vis the sequence of events, actors involved (i.e., oneself and others), facts, associated feelings, emotions, and sensations, and ecosystem variables. The reflection process may also include life implications, psychological (e.g., childhood experiences, attachment style) or spiritual (e.g., beliefs, rituals, identity, community) underpinnings, personal health, relationships, physiological shifts, symbolic imagery, and metaphor (Guss et al., 2020; Pernicano et al., 2022). The practitioner notices and surfaces the connection between the intention set and the content of the session, then expands on the elements that have emerged (Frymann et al., 2022).

##### Remorse

If the task of *Reckoning* was to acknowledge difficult truths and account for the harm, the task of *Remorse* is to metabolize those truths and emergent emotions, feelings, and sensations through “honest grieving.” Honest grieving allows a person to engage slowly with their inner, affective experiences and interoceptive network and become fully present to their moral pain by (a) allowing it to be felt; (b) recording the pain honestly, without hypocrisy, dishonesty, sentimentality, or idealization; and (c) recounting the pain directly. In doing so, new understandings about the pain are revealed, acceptance of the pain can be absorbed, and opportunities to transform the pain can be realized.

Despite the demonstrated importance for self-forgiveness of *Reckoning* one’s part in a meaningful transgression, the resulting emotions, feelings, and sensations can be all-encompassing (Buhagar, 2021; Cornish and Wade, 2015). For people suffering from MI, the habituation that can develop from analyzing a morally injurious experience has been shown to exacerbate feelings of pain, discomfort, vulnerability, exhaustion, disconnection, and overwhelm; it can even lead to suicidal ideation (Barnes et al., 2019; Litz et al., 2016; Nash, 2019). It is well documented that engaging with traumatic memories or difficult emotions can cause autonomic dysregulation (van der Kolk, 2014), such as hyper- or hypoarousal, which can impair the top-down cognitive functions of the prefrontal cortex, while intensifying the emotional responses of the amygdala and basal ganglia (Arnsten et al., 2015). It is also well documented that emotional processing has been shown to be effective in reducing or eliminating difficult emotions (Buhagar, 2021) and essential for self-forgiveness (Fisher and Exline, 2010; Griffin et al., 2015; Pernicano et al., 2022).

Nash and Litz (2013) have suggested that intense emotions, such as shame, guilt, and anger, may present impenetrable barriers for full disclosure from MI memories; moreover, that rarely do a person’s worst, most distressing experiences surface early in therapeutic sessions. Haidt (2003) proposed that emotions, particularly inner-directed negative emotions such as disgust, anger, guilt, and shame, direct moral judgment, choices, and beliefs, including harsh judgments about oneself, others, and the world. Such intense emotional experiences are similar to what Luskin (2003) has referred to as *core wounds*, that is exceptionally deep, if not suppressed, and intense pain that has created internalized messages about what a traumatic event or series of events meant to a person. He has argued that these emotional wounds are based on interpretation, especially those aimed at oneself, such as “I’m bad,” “I’m a failure,” “I’m powerless,” “I’m worthless,” “I’m inferior,” “I’m damaged,” “I do not deserve,” “I’m not enough,” “I’m all alone,” “I’m not wanted,” “I do not matter,” “I’m different,” “I’m wrong,” and “I’m not safe.” Over time, these negative self-concepts can manifest in entrenched patterns of behavior, reactions, and belief systems. Therefore, adequate pace (i.e., intensity, frequency, and duration of the writing engagement) and space (i.e., therapeutic support and writing environment) for the emotional processing of honest grieving are important if a person is to truly become present to their moral pain and absorb the impact of the violation.

Content activities for *Remorse* include intention setting and pre-session grounding exercises, which focus on identifying and metabolizing a client’s core wounds and internalized messages about their morally injurious experience—including the harm caused, values violated, beliefs questioned, perspective shifts, and relationship alterations or ruptures—by allowing for and titrating painful emotions, feelings, and sensations, the latter being a principle of trauma therapy (Briere and Scott, 2015) that appears to not have been used foundationally in other forgiveness or self-forgiveness approaches (Cornish and Wade, 2015; Enright, 1996; Griffin et al., 2015; Pernicano et al., 2022). Titration refers to how much emotional flow a person allows into their system. To titrate one’s experience is to keep themselves in an intentional place of choice and safety by opening and closing the tap on emotions. Titration is a process that slows down a person’s internal response—emotional, cognitive, and physiological—so that they can better maintain autonomic regulation, which allows for more effective information processing, rational thought, and meaning-making (Briere and Scott, 2015). Content activities also focus on clients accepting that painful emotions, feelings, and sensations are not inherently “bad,” as people often assume (Buhagar, 2021; Burkman et al., 2022; Pernicano et al., 2022); rather, they are neutral messengers with important information to share; likewise, that the pain one feels is a marker of their values still being intact. Intention setting and pre-session grounding exercises concentrate on negative “I” statements, such as those mentioned above, fostering self-compassion, recalling laments, and staying in one’s “window of tolerance” (Corrigan et al., 2011), that is an optimal arousal zone. Writing prompts include the “Mirror Test,” “Web of Life,” “Desire Inventory,” “Emotion Inventory & Assessment,” “Give Witness to Harm,” “Grievance Scan,” and “Open Your Heart” (DeMarco, 2024b). Integration can include reflections on the impact of the client’s core wounds, not only as they relate to their morally injurious experience, but also trace previous times when the person has experienced those same emotions, feelings, and sensations. This allows a person to bring better awareness and emotional clarity to the root of the issue. As with *Reckoning,* the reflection process may also include life implications, psychological (e.g., childhood experiences, attachment style) or spiritual (e.g., beliefs, rituals, identity, community) underpinnings, personal health, relationships, physiological shifts, symbolic imagery, and metaphor (Guss et al., 2020; Phelps, 2017). The practitioner notices and surfaces the connection between the intention set and the content of the session, then expands on the elements that have emerged (Frymann et al., 2022).

##### Reconcile

There is no clear normative standard for reconciliation. Definitions of reconciliation include both intra- and interpersonal approaches, such as rendering conflicted entities mutually consistent or coherent (Stanford Encyclopedia of Philosophy Archive, 2015); transforming relations among conflicted groups and rebuilding trust for a shared future (United States Institute of Peace, n.d., para. 1); a process that is focused on healing relationships (Cantacuzino and Karolyi, 2024); accepting an unpleasant truth or unavoidable situation and living better with the knowledge of that reality (Stanford Encyclopedia of Philosophy Archive, 2015); acknowledgment of past suffering and changing destructive attitudes and behavior into constructive and sustainable relationships (Brounéus, 2009); seeing the difference between two paths, and moving towards the one with broader vision (Burgess, 2022); transforming a person’s attitude so they can birth a new perspective (The Truth and Reconciliation Commission of Canada, 2015); and moving beyond civility and hidden resentment, to restoring love and trust (Jones, 2004).

As previously mentioned, whether and how reconciliation ought to be included as an element of self-forgiveness remains unclear (Enright, 1996; Hargrave, 1994; Strelan and Covic, 2006). Cornish and Wade (2015) omit reconciliation altogether. In their 4Rs model (2015), *Remorse* is followed immediately by *Restoration*, an action-oriented step focused on making amends with the offended person: “As remorse is expressed, the desire for restoration should emerge” (p. 98).

While more recent literature on self-forgiveness supports other-directed, reparative action as necessary for self-forgiveness (Bremault-Phillips et al., 2022; Buhagar, 2021; Cornish and Wade, 2015; Griffin et al., 2015), I suggest that for MI a mediatory or reconciling step—akin to a threshold between emotional processing and taking action—that focuses on trust building, ending hostility towards oneself, and restoring one’s sense of internal coherence through meaning-making must be included. Meaning-making is the process of how a person perceives, interprets, and makes sense of events in life, relationships, and themselves (Currier et al., 2015; Park, 2010). Meaning-making provides a way for people to organize memories and shape the narrative of an experience. It also helps to harmonize incongruities in one’s beliefs, expectations, and attitude toward life (Currier et al., 2015; Park, 2010). Researchers (Park, 2022; Wrosch, 2010) cite two types of meaning: global meaning and situational meaning. Global meaning refers to a person’s general orientation to life, such as overarching beliefs, goals, a sense of purpose, and assumptions about oneself, others, and the world. For instance, the belief that God is benevolent or that life is fair; that others are good or evil at heart; or that life is random or else determined. Situational meaning refers to how global meaning affects a person’s reaction to a certain situation. When global meaning and situational meaning reinforce each other, then a sense of coherence and trust develop (Galletta et al., 2019; Gifford, 2013). When these two types of meaning become incompatible, such as with MI, a sense of incoherence and distrust emerges (Currier et al., 2015; DeMarco, 2024a; Park, 2022). To regain coherence and trust after a morally injurious experience, one must reconcile—or harmonize—the experience and then reconsider or readjust how they understand one of the two, or both types of meaning; otherwise, any subsequent reparative action runs the risk of being inauthentic or truncated—or what could be called “cheap amends.”

Therefore, I present the third component of the 6-FPSF as *Reconcile*, a psychospiritually situated engagement that addresses internal dissonance, brings an end to hostility towards oneself, and comes into harmony with others and the world. This includes broader existential inquiry, accepting contradictions and imperfections, overcoming self-deception, right-sizing responsibilities, expanding one’s sense of self and self in context, considerations and reconsiderations about explanations for the transgressive event(s), reassessing values that were violated, fostering compassion for oneself, embracing common humanity, and finding “kernels of truth” (DeMarco, 2024a, p. 92). *Reconcile* is approached with “dynamic balance” that is maintaining a flexible, open, compassionate, and accepting posture so painful memories can heal—not by eliminating the memories, rather by integrating them into life’s larger story, and making oneself available to “a moment of grace.” In doing so, bonds of trust, self-worth, and meaning can begin to be restored.

Content activities for *Reconciling* include intention setting and pre-session grounding exercises that focus on self-awareness such as broadening one’s sense of self and worldview, global and/or situational meaning, metaphor, and ritual. Writing prompts include “Values Reset,” “Bittersweet,” Landscape of Life,” “Feel to Forgive, Reconcile to Restore,” “Vital Moments” and “Deconstruct Your Story” (DeMarco, 2024b). Integration for *Reconcile* can include reflections on the limits of responsibility, perceived and excessive responsibility, effects of core wounds, accepting oneself as being fallible but inherently worthy, honoring the morality of the transgression as wrong but also the transgressor as capable of doing good, barriers of beliefs, and assessing internal and external resources for healing. As with the first two components, the practitioner notices and surfaces the connection between the intention set and the content of the session, then expands on the elements that have emerged (Frymann et al., 2022).

#### Phase 2: soul remaking

*Soul Remaking* focuses on active self-expression and external engagement.

##### Rectify

As previously mentioned, researchers (Bremault-Phillips et al., 2022; Breines and Chen, 2012; Carpenter et al., 2014; Cornish and Wade, 2015; Fisher and Exline, 2010; Griffin et al., 2015; Nash and Litz, 2013; Woodyatt and Wenzel, 2013a,b) generally agree that self-forgiveness requires interpersonal engagement, which centers on making amends and reparative actions to those who have been harmed. Similarly, Carpenter et al. (2014) found that the more amends are made, the more people felt self-forgiveness was morally permissible. Exline et al. (2011), however, have suggested that self-forgiveness should not be encouraged until after reparations are made, likely because people felt more deserving. Wenzel et al. (2021) and Buhagar (2021) agree that making amends is an important part of regaining moral integrity after a transgression, but they acknowledge that in some cases this may be both undesirable and impossible, such as when the person(s) wronged are either unavailable or unwilling to accept direct amends or when those amends may cause further harm. In these instances, it has been proposed that modifying one’s own attitude and beliefs can serve the reparative purpose; also, committing to living in a way that reflects what the intention of the amends might have been—or what Burkman et al. (2022) refer to as “living amends” (p. 108). Other authors have also discussed the importance of addressing a person’s attitudes, beliefs, and behavioral patterns that contributed to the transgression (Baker, 2008; Holmgren, 1998); likewise, that they recommit to the values that were violated (Bremault-Phillips et al., 2022).

Some authors have referred to making amends as a “restoration” stage (Cornish and Wade, 2015). Meanings of *restore* include reinstating something former, turning back, and returning to an original state or condition (Online Etymology Dictionary, 2024). As will be discussed further in the following component, I suggest that for significant transgressions, such as MI, it is neither enough nor possible to “go back” to what previously existed in its “original” form, whether that is a relationship, perspective, assumption, expectation, or way of being. This is because once a violation occurs, it changes those events, people, and relationships that created the initial violation (DeMarco, 2024a; Lederach, 2015)—but violations can be rectified. Definitions of *rectify* include “to correct something” (Cambridge Dictionary, n.d., para. 1); “to improve something” (Vocabulary.com, 2024); and an “essential changing to make something right, just, or properly controlled or directed” (Merriam-Webster, 2024). This last definition is especially resonant to the proposed concept: specifically, that while moral violations leave an indelible mark on the human mind and soul (Brock and Lettini, 2012), those marks are a meaningful part of the person’s story and ought to serve as the foundation, even inspiration for healing and a life of integrity going forward.

Therefore, I present the fourth component of the 6-FPSF as *Rectify*, an action-oriented, behaviorally situated engagement that addresses both the episode (i.e., direct harm from the specific violation) and the epicenter (i.e., essential or systemic attitudes and beliefs that grew out of negative past patterns and experiences) of the moral injury to create constructive change processes that move a person from self-estrangement to self-engagement, that is being more comfortable with themselves, others, and the world. *Rectify* includes righting relationships; making amends (as possible) or living amends (Burkman et al., 2022); attending to problematic behaviors and habits, such as rumination, self-harm, and risky behavior (Barnes et al., 2019; Farnsworth et al., 2014); articulating new parameters for healthy connection, participation, and expression of values; opening new pathways for bonding and belonging; and adopting new ways of thinking and being. *Rectify* is approached with “positive intention,” that is with the understanding that the transgressor is “acting in good faith”—with honest desire and purpose, pure motives, and goodwill—doing the best they can. Good faith is a mark of moral integrity:

“…the person no longer holds himself at all apart from the desire to which he has committed himself … The decision [to act] determines what the person really wants by making the desires upon which he decides fully his own. To this extent the person, in making a decision by which he identifies with a desire, *constitutes himself*” (Stanford Encyclopedia of Philosophy, 2019, p. 38).

By constituting oneself without ambivalence or inconsistency, one has wholeheartedness, which is equated to integrity (Stanford Encyclopedia of Philosophy, 2019).

Content activities for *Rectify* include intention setting and pre-session grounding exercises that focus on expressing penitential feelings, acknowledgments, or beliefs to the person or entity that was harmed, as it would be safe to do so; considerations for offering a gift or ritual if making amends is not possible; implementing new boundaries with self, others, patterns, places, experiences, work, God/Divine, community, and the world; finding renewed purpose; committing or recommitting to core values; growing in connection; building resilience; and experiencing transcendence, the latter understood as a sense of wonder and awe (Wulff, 1997). Writing prompts include “Turning Away from Fear/Shame/Guilt’s Voice,” “Preluding a Moment,” “Habits of Harm, Habits of Heart,” “Positive Intention,” and “Words Made Real” (DeMarco, 2024b). Integration can include reflections on attitudes and beliefs that grew out of negative past patterns and experiences; past and present emotional, psychic, and spiritual boundaries; requirements for reconnection; insights about integrity; preparing to receive the wronged person’s response with benevolent honesty and grace; and internal and external resources, including safe contacts.

##### Re-creation

Some researchers (Cornish and Wade, 2015; Buhagar, 2021) maintain that once amends or restorative actions are made, the offense can be resolved, making way for the final component or typical end-state of self-forgiveness. Cornish and Wade (2015) refer to this as “renewal.” To hold onto negative emotions about the offense, it has been suggested, would serve no functional purpose (Holmgren, 1998). Instead, the transgressor recognizes their intrinsic worth as a person with kindness, compassion, acceptance, and respect—full stop (Cornish and Wade, 2015).

Resolution implies that a matter is settled, in the case of MI that the “injury story” is complete, and yet other researchers (Bremault-Phillips et al., 2022; Burkman et al., 2022) suggest that self-forgiveness is ongoing/persistent in nature, thus requiring an ongoing process. The final sessions of Impact of Killing in War (IOK; 2022), a cognitive-behaviorally-based treatment that focuses on self-forgiveness and moral repair after killing, includes transgressors (i.e., veterans) assessing what has changed since the beginning of treatment and what areas remain conflicted, cautioning that some aspects of their experiences may never fully be resolved. Other authors (Litz et al., 2016; Pernicano et al., 2022) agree that there is no “one-and-done” treatment for healing MI. While I agree, I also share the view with those (Antal and Winings, 2015; Buhagar, 2021) who suggest that self-forgiveness is an ongoing journey or pilgrimage—that the “injury story” does not end with the cessation of hostility towards oneself and reparations for those wronged. Indeed, while the impulse to “resolve” can lead to providing short-term relief to pain (Lederach, 2015), it also runs the risk of false or forced self-forgiveness, which may only add to the moral injury (Burkman et al., 2022).

Therefore, I present the fifth component of the 6-FPSF as *Re-creation*, an embodied, situated engagement that focuses on transformation, a process whereby a person shifts holistically from a self-corrosive, self-alienating sense of self (Bremault-Phillips et al., 2022) to one that is self-accepting, self-expressive, and honors their intrinsic self-worth as a “sacred self” (DeMarco, 2024b, p. 147). Such transformation results from developing a *coherent sense of time* (DeMarco, 2024a), that is seeing oneself fully in the past, present, and future with equal value; also, by altering the “injury narrative” to advance a new story that integrates *all* aspects of experience, with an expanded perspective of self, others, and the world. This includes a person’s painful experiences, as sources of wisdom and guidance.

“When we transform something, we evolve one thing into another. What is important to remember is that the thing that once was does not simply go away; a seed no longer looks like a seed once it is planted and in full bloom. Its “seedness”—its essence remains embedded in the new growth” (DeMarco, 2024a, pp. 158–159).

Through *Re-creation* a person acknowledges that while they may not be able to change the moral rupture of their past, they can still choose to act in the present and adapt their core self and values for the future.

*Re-creation* can be likened to *kintsugi,* the Japanese art of transforming broken pottery by repairing it with powdered gold, silver, and platinum (Richman-Abdou, 2022). At its core, kintsugi views broken objects as not inherently worthless; indeed, every fractured piece brings meaning to the life of the object. By creating something new and beautiful, the piece is made more valuable and whole and extends its purpose rather than ceasing it at the time of damage. Emphasizing the cracks can be symbolic for embracing the imperfection of human existence. The breaks, knocks, and shattering of that which is beautiful, meaningful, or sacred need not be an end or a death; rather, they can be a new beginning for something good and true. Acceptance and Forgiveness Therapy (AFT; Pernicano et al., 2022) a psychospiritual group intervention for MI utilizes the “Cracked Glass Bowl” (Pernicano, 2014) exercise, seemingly modeled after kintsugi. Brock (2018) has also discussed kintsugi as a metaphor linked to MI.

*Re-creation* is approached in the spirit of kintsugi, that is affirming the value of all experience, the continuance of one’s journey of self-forgiveness beyond resolution to transformation, and that one’s integrity can still be found—or resurrected or re-created—not despite, but because of moral harm and “brokenness.”

Content activities for *Re-creation* include intention setting and pre-session grounding exercises that focus on attending to personal growth, honoring one’s sense of self-worth and wholeness, and reclaiming, re-creating, and sustaining important relationships and connections. Writing prompts include “Revisioning Relationships,” “Gentle Heart,” “Three ‘S’ Selves,” “Symptom Symbols,” “Accepting Risk,” and “Your Calling, Your Purpose” (DeMarco, 2024b). Integration can include reflections on growing acceptance and self-forgiveness, interpreting “cracks,” viewing the person’s “injury story” and nested, that is as a story within a series of stories that make up the story of life, creating new “healing story lines,” what personal growth looks like now and commitments to it, and metaphors of transformation; for example, the ancient, fabled mythic phoenix who continues to rise from the ashes anew.

##### Remain

Rushton (2018) introduced the concept of *moral resilience*, that is the capacity a person has to restore or sustain integrity when faced with moral adversity, in response to increasing reports of moral distress among healthcare providers. While still a nascent concept in need of refinement (Young and Rushton, 2017), moral distress has broadened its application to include other professions, such as medical professionals generally, social service providers, teachers, law enforcement, the military, emergency service providers, lawyers, journalists, and politicians, among others. In one’s day-to-day life, this type of suffering can take a meaningful toll on beliefs, relationships, and affiliations (Feinstein and Osmann, 2023; Jaskela et al., 2018).

As with resilience, generally, moral resilience may be viewed as a *way of being*, understood as authentic inquiry, reflection, and aligned action that endures with the evolving nature of the self and self in the world (Halonen and Lomas, 2014). Moral resilience involves building, nurturing, and sustaining a person’s capacity to navigate moral adversity and developing systems that support an ethos of integrity (Rushton, 2018). Moral resilience is grounded in *moral conscientiousness*, that is a vigilance to live in ways that are aligned with who a person is at their core and what they stand for amid situations that appear in contrast with integrity (Rushton, 2016). Moral resilience also involves *ethical embodiment* (Rushton, 2016), that is living the values that a person espouses through their actions and decisions and cultivating a moral vocabulary, imagination, attitude, coherent character, and dynamic moral posture. Moral resilience further involves speaking with clarity and confidence by attending to one’s moral concerns as they arise, in the appropriate settings, and with invested others (Rushton, 2016). To view self-forgiveness as a journey, rather than a destination or a point on a spectrum to reach, as earlier suggested, is to acknowledge the perennial nature of self-forgiveness, with moral resilience at its core.

Therefore, I present the sixth and final component of the 6-FPSF as *Remain*, also an embodied, situated engagement that focuses on sustaining the trusting and worthy *sacred self* that was re-created in component #5 and is subsequently active in the world. *Remain* allows a person to embrace self-forgiveness as *a way of being*—the long-term process of living the new, coherent, values-driven story from *Re-create*. Through *Remain* a person abides in conscience, character, choice, commitment, community, and contribution. They appreciate the journey to here and beyond. They accommodate twinges of negative emotions and rumination that can naturally arise (Burkman et al., 2022) and reset by finding meaning, purpose, value, connection, and transcendence. They attune their values on an ongoing basis, practice ethical embodiment, and immerse themselves in the “moral world” by cultivating moral conscientiousness (Rushton, 2016). *Remain* is approached with trust or faith, faith being understood as affirming, aligning with, and abiding in something without epistemological certainty (Dyess, 2011; Stanford Encyclopedia of Philosophy, 2019). Life is imperfect; a person cannot predict future events or experiences; therefore, it cannot be certain that the transgressor will not transgress their moral values again, but they can practice moral resilience and revisit the 6-FPSF if necessary.

Content activities for *Remain* include intention setting and pre-session grounding exercises that focus on fostering personal integrity, relational integrity, and patience in the face of moral adversity, struggling well, maintaining a dynamic moral posture, and moral efficacy. Writing prompts include “Healing Haiku,” “Sensing Soul,” “Anticipatory Savoring,” “I am Here, I Am Ready,” and “Express Yourself” (DeMarco, 2024b). Integration can include reflections on wise decision-making, moral attitudes and behaviors, overcoming disappointment and failure, setting aside lingering self-condemnation, accepting human limitations, lessons learned, positive change gleaned from the self-forgiveness process to this point, and forgiving not forgetting.

## Discussion

MI is gaining increasing attention from researchers and practitioners who consistently call for effective treatments that differ from those approaches commonly used with PTSD (Bremault-Phillips et al., 2022) and that overcome barriers to cost, accessibility, and stigma (Griffin et al., 2015). Litz et al. (2009) have argued for the development of interventions that help clients cope with self-blame, self-condemnation, and negative emotions, such as guilt and shame. Bremault-Phillips et al. (2022) have shown how forgiveness practices may help to restore one’s sense of and relationship with self, others, and the Sacred and to nurture reconciliation and healing. Bremault-Phillips et al. (2022) and others (Buhagar, 2021; Nash and Litz, 2013) further suggest the importance of understanding forgiveness from an interdisciplinary perspective and recommend greater development of novel, interdisciplinary approaches. Burkman et al. (2022) have discussed self-forgiveness as an ongoing healing process beyond making amends. Presented in this study is the 6-FPSF, an interdisciplinary, narrative-based conceptual model for the treatment of MI, particularly self-induced MI, based on self-forgiveness, and is one solution that may meet these vital clinical needs.

Given that MI can have a very powerful embodied impact, particularly among those with self-directed MI, the construction of a model for self-forgiveness is both significant and timely. Studies (Corona et al., 2019) have consistently found more severe suicidal ideation in individuals experiencing self-induced moral transgressions. Similarly, military men who reported PMIE exposure by perpetration were 50% more likely to attempt suicide during service and twice as likely to attempt suicide after separating from service (Corona et al., 2019).

Given that MI is still a nascent but quickly growing field, and that there is a paucity of interventions that have proven effective, it is unlikely that specialized training in MI will keep up with the public demand. Accordingly, it is reasonable to anticipate that many unidentified cases of MI will likely result. This may be compounded by the current tension between mental health providers and spiritual/religious leaders who wrestle with definitions of MI, the underlying mechanisms of MI, whether MI ought to be included in the *DSM-5*, and what the best approach to treatment is (Bremault-Phillips et al., 2022; Litz et al., 2016). It is essential that the scientific community better understand the processing of self-directed MI and self-forgiveness practices, so that practitioners and clients can administer interventions that best address MI’s multidimensional nature and maximize sustainable benefits of self-forgiveness. The 6-FPSF has the potential to forward both the multidimensional concerns and maximization of benefits.

### Limitations and future directions

Although I have informally treated more than 15 clients using the 6-FPSF with positive evaluations, the model has not been empirically tested. Future research, such as larger controlled studies and clinical experimentation is needed to draw reliable conclusions. For instance, comparing treatment results and clinical experiences for clients who were treated by an individual therapist versus clients who were treated using the 6-FPSF, or other forgiveness models such as Cornish and Wade’s (2015) “4Rs” model, and models for MI specifically, such as IOK (Burkman et al., 2022), ADT (Litz et al., 2016), or Acceptance and Commitment Therapy for Moral Injury (ACT-MI; Borges, 2019). Whether, or the extent to which, the inclusion of reconciliation, as an element of self-forgiveness, makes a meaningful impact on healing MI remains unclear and ought to be tested; similarly, is the relationship between self-forgiveness and moral resilience (see *Remain*). Several of the clients who were informally treated had comorbidity with PTSD, which should be considered in the exclusion criteria.

The most efficacious number of sessions remains unclear given the complex nature of self-forgiveness and the inclusion of integration in addition to writing. Of the informal treatment of the 6-FPSF that I undertook, clients generally reported benefitting most from 12 sessions for 60 min each (except for the first which was 90 min, allowing for psychoeducation). Several reported favoring two sessions per week because it provided a more immersive experience. Whether there is a significant difference in outcome between 6 weeks with two sessions per week or, for instance, 12 weeks with one session per week is unclear and ought to be tested; likewise, is whether increasing the number of sessions (e.g., to 24). Several clients reported desiring more time and space for writing, reflection, and integration. The issue of whether the 6-SPSF can be successfully administered in a group setting is another area of inquiry. I held one informal group (of five participants) who reported strong positive experiences; whether the efficacy of the 6-SPSF is significant in a group setting also bears research, as well as whether one mode is more successful than another. It is worth mentioning that in the one informal group, I extended the length of the session to 90 min for each with the exception of the first group which I increased to 120 min to allow for the greater number of people during integration.

Whether the practitioner should have specialized training—in trauma generally, or MI specifically—also remains unclear. While I have no information about the level of training and experience needed to successfully implement the 6-FPSF, I have hypothesized that a foundational understanding of the concepts, theories, and practices of both would be necessary, particularly because of the clinical skills required for integration.

The question of whether integration makes a measurable impact also bears consideration. I have also hypothesized that making sense of and finding significance in the writing experience, discovering insights gleaned during the session, and applying them to the process of self-forgiveness going forward would be especially helpful, given the importance of meaning-making in healing MI. In my informal application of the 6-FPSF, clients generally reported positive experiences from the integration. This is an important issue to explore in future research, not the least of which is because it impacts the time and effort it takes to implement the model. Given the increasing number of individuals in need of treatment services for MI, many of whom do not present at mental health treatment centers, it is critical to develop interventions that can be easily disseminated and implemented by a broad group of professionals.

Lastly, the implementation of the 6-FPSF should also be included for future investigation. It would be valuable to know why practitioners choose the 6-FPSF and how they might modify the intervention. Such alterations could contribute to routine care, which may lead to greater efficacy of treatment.

## Conclusion

Given the increasing number of individuals in need of treatment services for MI, many of whom do not present at mental health treatment centers, it is critical to develop interventions that can be easily disseminated and implemented by a broad group of professionals. Emerging research on MI consistently shows its multidimensional nature, thus requiring an interdisciplinary, holistic approach for healing (Bremault-Phillips et al., 2022; Burkman et al., 2022; Hodgson and Carey, 2017; Kinghorn, 2012; Litz et al., 2009; Pernicano et al., 2022). It is vital that treatment options extend beyond traditional protocols for PTSD, as they have been shown to be ineffective for MI (Steenkamp et al., 2015), likely because of differences between the two conditions (e.g., the clash between a person’s conscience and overwhelming existential experiences, spiritual distress such as acute loss of one’s faith and erosion of meaning and purpose, appropriately resolving guilt/blame/shame, and self-forgiveness). The 6-FPSF fills this vital need by addressing the psychological, emotional, behavioral, social/relational, somatic, and spiritual elements of moral trauma (Kinghorn, 2012) and the intra- and interpersonal aspects necessary to foster moral repair (Cornish and Wade, 2015) and moral resilience (Rushton, 2016). The 6-FPSF protocol was designed to facilitate self-forgiveness among those with MI and may be a valuable tool for reestablishing essential bonds of self-worth, trust, and life-sustaining relationships.

## Data Availability

The original contributions presented in the study are included in the article/supplementary material, further inquiries can be directed to the corresponding author.
